# Situation Awareness in Remote Operators of Autonomous Vehicles: Developing a Taxonomy of Situation Awareness in Video-Relays of Driving Scenes

**DOI:** 10.3389/fpsyg.2021.727500

**Published:** 2021-11-10

**Authors:** Clare Mutzenich, Szonya Durant, Shaun Helman, Polly Dalton

**Affiliations:** ^1^Royal Holloway, University of London, London, United Kingdom; ^2^Transport Research Laboratory, Crowthorn, United Kingdom

**Keywords:** situation awareness (SA), SA comprehension, SA Prediction, driving, video, taxonomy

## Abstract

Even entirely driverless vehicles will sometimes require remote human intervention. Existing SA frameworks do not acknowledge the significant human factors challenges unique to a driver in charge of a vehicle that they are not physically occupying. Remote operators will have to build up a mental model of the remote environment facilitated by monitor view and video feed. We took a novel approach to “freeze and probe” techniques to measure SA, employing a qualitative verbal elicitation task to uncover what people “see” in a remote scene when they are not constrained by rigid questioning. Participants (*n* = *10*) watched eight videos of driving scenes randomized and counterbalanced across four road types (motorway, rural, residential and A road). Participants recorded spoken descriptions when each video stopped, detailing what was happening (SA Comprehension) and what could happen next (SA Prediction). Participant transcripts provided a rich catalog of verbal data reflecting clear interactions between different SA levels. This suggests that acquiring SA in remote scenes is a flexible and fluctuating process of combining comprehension and prediction globally rather than serially, in contrast to what has sometimes been implied by previous SA methodologies (Jones and Endsley, 1996; Endsley, 2000, 2017b). Inductive thematic analysis was used to categorize participants’ responses into a taxonomy aimed at capturing the key elements of people’s reported SA for videos of driving situations. We suggest that existing theories of SA need to be more sensitively applied to remote driving contexts such as remote operators of autonomous vehicles.

## Introduction

Recent years have seen a transition toward ever-increasing levels of vehicle autonomy. With the aim of providing a universal taxonomy for defining levels of automation, the organizational body SAE International (2016) highlights six levels of automation for on-road vehicles. At the final stage of this taxonomy the car is fully self-driving, and the occupant is never required to take over. Yet even entirely driverless vehicles of this type will sometimes require human intervention. For example, an autonomous vehicle (AV) is unlikely to be able to interpret a contractor directing traffic using hand signals at a construction site, whereas a human can do so easily. In “edge” cases such as this, it is likely that human operators will step in to interpret the unexpected situation, and their input will need to be provided remotely in vehicles with no “backup” driver present at the scene.

The established understanding of automated driving is being continuously updated and now recognizes the need for occasional remote operation even at the higher levels of automation (Mutzenich et al., 2021). The industry standard taxonomy, SAE J3016, which outlines and defines the modes of automated driving was further updated in April 2021 to include remote support functions (SAE International, 2021). It is likely that, in some circumstances, a remote operator (RO) may even need to take over the real-time driving of the car (for example, if the automation has completely failed). Our research sets out to address the significant human factors challenges unique to a remote operator temporarily in charge of an automated vehicle.

For ROs to build up a mental model of the environment that they do not physically occupy, they will need a rich array of information from a variety of different perspectives to help them. Most remote driving is facilitated by monitor view and video feed, which is limited in terms of the size of the view presented and the reliance on 2D depth cues, making it inevitably second-rate to being physically present inside the vehicle. Yet the differences in building and maintaining remote SA have historically received relatively little attention in a road transport context and certainly not in remote operators of automated vehicles. How SA is attained from video relay is an important consideration when designing remote operator interfaces and training programs.

An RO who has been alerted by an AV to “drop in” and assess the problem or assume direct driving control will first need to acquire situation awareness (SA) of the remote scene. SA encompasses what is known about the environment, what is happening in it and what might change. There are many definitions of SA (see Gugerty, 1997, 2011; Endsley et al., 2003; Niklasson et al., 2007; Endsley, 2015; Lo et al., 2016) but the most cited is from Endsley’s model which divides knowledge of the environment into three levels of awareness: perception, comprehension and projection. However, the concept of projection (Level 3 SA) typically includes factual calculations not within the domain of the average driver (note that Endsley’s original research used military pilots adept at interpreting data from multiple instruments). We argue that “prediction” is a better descriptor for this level of driver SA, as predictions are based on subjective analysis of known external factors, which may change and are uncontrollable, but nevertheless appear likely to happen on the basis of the driver’s general experience. Therefore, we refer to the levels of SA simply as perception, comprehension and prediction (rather than projection).

Endsley’s original SA model was developed using military pilots but attempts have been made to extend it to *in situ* driving contexts (Ma and Kaber, 2005; Bolstad et al., 2010; Endsley, 2019). Further models of SA have been developed which align more closely with the driving domain than Endsley’s (Matthews et al., 2001). Matthews’ SA model (2001) divided driving awareness into strategic, tactical, and operational goals, putting the emphasis on the driver’s objectives. Strategic driving is ultimately goal orientated, such as destination and route, which may be part of existing mental models. Tactical driving is conscious and intentional, involving decisions such as when to overtake or change speed which require feedback from the environment and system. Operational driving implements tactical decisions into dynamic driving actions and maneuvers such as steering wheel control or braking. These driving goals interact to some extent and can also map onto Endsley’s three levels of SA in varying degrees, for example strategic driving is mostly Level 3 Projection, but Level 1 Perception and Level 2 Comprehension are also integrated.

Research that has considered the functional role of SA in automation and driving has so far been limited to the SA level of an *in situ* driver who has to take over the driving of an AV that can drive alone for short periods of time, for example measuring the response times of drivers assuming command after adaptive cruise control has been employed (Banks et al., 2018); investigating the issues of complacency and overtrust when humans are required to be in an active monitoring state for prolonged periods of time in automated cars (Larsson et al., 2014) and the current trends in designing automated vehicles which influence *in situ* driver’s SA (Walker et al., 2008). However, the prospect of ROs occasionally having to unexpectedly take control of AVs from a separate location makes it essential to identify how their SA needs will be different from those of an on-site driver.

Neither Endsley’s nor Matthews’ SA models tell us much about how people use the information in their environment to build SA. Some researchers have considered whether it is appropriate to apply Endsley (1995) model of SA to driving at all, querying whether it is more appropriate to refer to SA as cyclical, demonstrating top down and bottom processing rather than constructed *via* hierarchical levels (Neisser, 1976 as cited in Revell et al., 2020). Salmon et al. (2012) conducted a comprehensive review of SA and techniques and methodologies for assessing SA, arguing in favor of a systems-based explanation that considers the cycle of activity encompassing all road users together with the road environment and infrastructure, other vehicles and the impact of developing technologies combined. In which case, the question of how to effectively measure remote operator SA represents an operational challenge in the field.

Across the literature, the Situation Awareness Global Assessment Technique (SAGAT), a quantitative measurement of SA, is widely accepted to objectively measure SA (Endsley, 1988). SAGAT is a freeze and probe technique, where simulated trials are halted, and participants asked questions designed to measure SA levels of perception, comprehension and projection. Their responses are then compared with the reality of the simulation in order to derive a performance score. Previous attempts to measure SA in driving may lack construct validity as they are based on *a priori* notions of what driver SA “is” in the mind of the researcher. Studies that have adopted SAGAT to measure driving SA have typically measured people’s awareness in a range of different ways e.g., using a different number of probes or trials (Scholtz et al., 2005; Ma and Kaber, 2007); using a reconstruction task or a recognition task (Gugerty, 1997; Franz et al., 2015); or using a verbal recording in real time (Endsley, 2017a). Pre-determined questions are likely to artificially constrain the level of SA that participants are able to demonstrate (e.g., a person may be continually aware of the weather, but if they are not probed about this awareness, they will not be able to demonstrate this awareness) and, combined with the simulated nature and variability of the task set, do not get at the heart of what people “see” in their environment when viewing a remote driving scene. Nevertheless, we acknowledge Endsley, (2015, p. 9) argument that SA is part of “*deeply embedded mental models and schemas*,” and that the quantitative methodology that SAGAT adopts is necessary to extract information that people would otherwise be unable to communicate because they cannot be relied upon to have insight into their own SA.

### Outline of the Current Study

We contend that it seems likely that some combination of qualitative and quantitative approaches will eventually provide the fullest understanding of remote driving SA. However, as a starting point perhaps the most direct technique to get at this abstract and conceptual data is simply to ask **what** people see in a remote scene and capture and interpret every detail of what they report. Given that remote driving SA is such a new field, it makes sense to start from an unconstrained tool to inspect the implicit and explicit mental processes that are underway while ROs build up a remote model of the environment. With this aim, the current study made use of verbal elicitation techniques to uncover, in its most basic form, what people “see” in a remote scene when they are not constrained by rigid questioning. Video elicitation methods which require videos to be narrated by an expert as to their thinking, decision making and interpretation of the stimuli, have been found to be helpful in identifying “invisible phenomena” that are difficult to abstract using quantitative methods (Jewitt, 2012, p. 4). Likewise, retrospective verbal protocol techniques which encourage participants to provide a commentary of information they drew from the environment and what they were thinking about it have been shown to measure the cognitive processes that support SA Perception and SA Comprehension and the feedback process that shapes SA Prediction (Walker et al., 2008). ROs will need to take decisions based on the SA that they have conscious access to, so freely elicited verbal description seems just as likely to get at deeply embedded mental models and schemas as a SAGAT probe.

Operators will have to build up a mental model of the remote environment facilitated by monitor view and video feed, meaning the task of developing SA from a remote location is likely to be made more difficult by the operator’s physical absence, as well as any signal degradation. A comprehensive inventory of the mental models that underpin the construction of driving SA from video feeds is thus a clear research priority as video will surely play a role in remote operation. In this study, we used real footage filmed from a driving perspective, giving a naturalistic experience of a remote driving situation. We investigated how participants build up mental representations of these naturalistic remote driving scenes, using a verbal elicitation protocol at the end of the video. We also examined whether providing extra information from the rear-view camera footage influenced this process. Inductive thematic analysis was used to encode participants’ responses into a taxonomy aimed at capturing the key elements of people’s reported SA for videos of driving situations.

### Justification of Method

Inductive thematic analysis (TA) is the practice of encoding qualitative data by searching across the whole raw data set to find recurring patterns, regarded as “themes,” which are then used to generate a theory (Braun and Clarke, 2013). This type of analysis is likely to be less affected by researchers’ *a priori* preconceptions of SA than deductive TA which searches for evidence in datasets of pre-existing theories (e.g., in the context of our research, Endsley’s three “levels” of SA would have been the most obvious candidate for a pre-existing theoretical framework; (Boyatzis, 1998)). This paper adopts an inductive TA methodology to investigate more freely the complex processes that generate SA construction in remote viewing, which are not presently identified in quantitative metrics, generating deeper understanding of the semantic information that people can access in naturalistic driving scenes.

The inductive TA procedure in this paper followed the phases of thematic analysis outlined in Braun and Clarke (2013). Firstly, the lead researcher transcribed all participant verbal responses to familiarize herself with the data, then initial codes were generated using a data driven approach by listing all words used by each participant. Themes were developed using a semantic approach which reports the explicit meaning of codes, rather than a latent analysis which seeks to identify underlying meanings or assumptions behind the codes (Boyatzis, 1998). Themes were then grouped into patterns and reviewed by checking that they were mutually exclusive, and that the entire data set could be classified into the suggested themes. Finally, themes were named and defined according to how they best described the features of that theme. During the process, we assumed an active role in determining themes, reflected in our analysis where, rather than reporting themes as passively “emerging” from the data, which has been the subject of much criticism in this methodology, we are careful to provide sufficient detail in the process of determining the coding decisions that were made in respect to item definition and inclusion in the construction of themes (Braun and Clarke, 2013, p. 80).

The analysis of participant transcripts using this approach enabled the construction of a taxonomy of SA in video-relays of driving scenes. Taxonomies serve to classify concepts and give insight into the principles which underlie these classifications. Previous taxonomies relating specifically to driving have mainly focused on driving errors and violations to identify causal factors and risks involved on different highway and environmental contexts (Stanton and Salmon, 2009; Khattak et al., 2021) or in highly automated cars, the factors that lead to handovers in critical situations (Capallera et al., 2019) whereas our taxonomy delineates the mental models that underpin the construction of driving SA *via* video, facilitating an understanding of the themes that make up someone’s remote SA. Once validated, it is anticipated that this taxonomy can be used to develop regulatory frameworks for training remote operators of AVs and will be used in future empirical work in our laboratory to design quantitative queries that can effectively measure the SA of ROs.

## Materials and Methods

### Developing the Driving Video Stimuli

**FIGURE 1:**
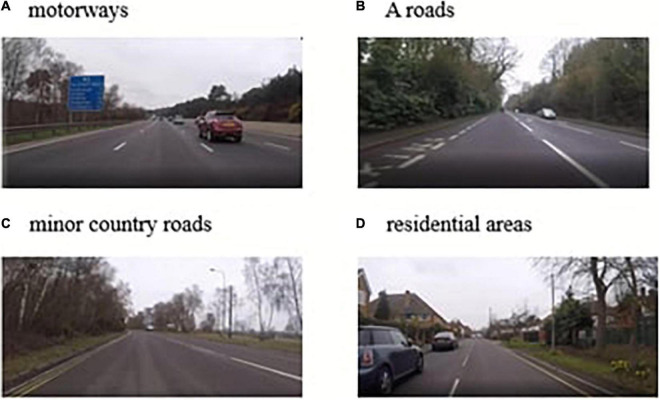
Examples of different road types: **(A)** motorways, **(B)** a roads, **(C)** minor country roads, and **(D)** residential areas.

To create the driving videos, researchers drove a 2016 petrol Dacai Duster 4 × 4 (manual right-hand drive) for 32 min filming continuously. The 21.8 miles route was from Royal Holloway, University of London, Egham, using the following roads, A328/B329/A30/M3/A331/A30. The route was designed to encounter a range of roads covering all speed limits and including motorways, A roads, minor country roads and residential areas (see Figure 1 for examples of different road types). This was in line with previous research that had created videos of on-road driving scenes (Walker et al., 2008; Salmon et al., 2013). This paper aims to address the lack of standardization in measuring driving SA by providing a complete, open-source set of remote videos of driving scenes^1^.

Video and audio recordings of the forward and rear views were filmed using two GoPro6 cameras mounted using the GoPro Suction Cup Mount. The front camera was mounted 60 cm from the end of the bonnet and 50 cm lateral from the widest point to ensure that it was not obstructing the driver’s view and the end of the bonnet was not in shot. The rear camera was mounted 20 cm up from the bottom of the rear-view window and 50 cm lateral position pointing at the road behind the vehicle. This distance was selected because it did not obstruct the driver’s view and the body of the car was not visible. Both camera angles were verified from the driver’s position inside the car to confirm they accurately reflected the available forward facing and rear-view perspective. This gave a first-person perspective of the road ahead and pilot tests verified that it accurately reflected what the driver was seeing using in-car footage relayed to a mobile phone. Both cameras were controlled inside the car and recording started as soon as the driver released the handbrake.

**FIGURE 2:**
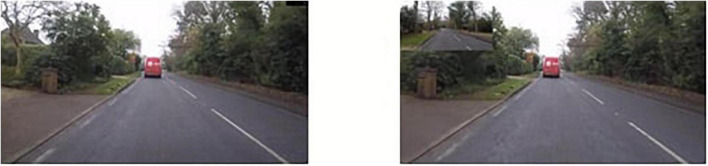
Images showing the two versions of the video, without (**left** image) and with a rear-view insert (**right** hand image).

The 32-min recording was edited using Camtasia©^2^ to create eight separate video segments divided into “A roads” (A30/331), “Residential roads” (A328), “Rural roads” (B329) and “Motorway roads” (M3). Two versions of each video were created; a forward-facing video recording of the total drive (“rear-view absent” condition) and a second version of the video with the addition of the rear facing footage positioned in the top left corner of the video (“rear-view present” condition) (see Figure 2 for images showing the two versions of the video). In this study, rear-view footage of the car was presented to the left of the center of the driving video to increase mundane realism, as it is typically the location of the rear-view mirror in a car in the United Kingdom (Buscher et al., 2009). The image was set at a ratio of 34.8% of the total image in the top left corner of the screen. This size was based on the approximate proportional scale of a rear-view mirror to the windscreen in a car, as there are no standard sizes for either mirror or windshield in United Kingdom regulations.

We used the Gorilla Experiment Builder^3^ to create and host our experiment (Anwyl-Irvine et al., 2018). Data was collected between 9th September 2019 and 7th October 2019. Participants were recruited through Prolific. Participants took part *via* a laptop or desktop computer, and we excluded the use of smartphones and tablets. Participants were advised that they must be in a quiet environment with no background noise or distractions. They were required to have a working microphone on their PC and completed a microphone audio test to proceed in the experiment. Videos were presented at 60 frames per second on an 811 × 456 pixel screen placement holder.

A preliminary study was conducted (*n* = *10*) to test the audio recording task in Gorilla and check the quality of the driving videos during online testing. During this testing procedure, it was evident that participants were unsure of what to say and there were large differences in the amount and quality of content that they gave in response to the questions with some comments not directly relevant to driving. Other research that has used qualitative “think aloud” methodologies or verbal protocols has provided participants with practice trials and/or suggested the kinds of items or events that could be appropriate to mention (Walker et al., 2008; Salmon et al., 2013). This could be viewed as pushing participants to say certain kinds of things and could therefore introduce bias. However, the limited range and detail of observations in the pilot transcripts suggested that an example trial was necessary in the current experiment, to ensure that participants gave sufficiently full accounts. For this reason, the final version of the experiment included an example trial, described in more detail in the “Procedure” section below.

During the preliminary study, we discovered that the road sounds that accompanied the videos were likely to provide extra situation awareness cues to aspects such as speed, possibly creating a more immersive experience. Some remote operation training teaches operators how to pick up cues from other feedback, for example spatial audio above, around and behind the car (Tang et al., 2013; Störmer, 2019; UNECE, 2020). Although participants in this study were unlikely to possess this type of skill, they may not have all had audio enabled on their computers, so we removed the audio track from the videos to reduce this source of variability between the experimental stimuli. The effects of the presence (vs. absence) of this audio is an interesting issue for future empirical studies, and some of this research is currently underway in our lab.

### Design

We adapted the SAGAT methodology to create a qualitative task. At the end of each video, participants were asked to respond verbally to two open questions: “what is happening?” (to determine their comprehension of the scene); and “what will happen next?” (assessing the ability to make predictions from their understanding of the scene).

We also examined whether providing extra information from the rear-view camera footage influenced this process. In a within groups experimental design, all participants watched four videos with a rear-view mirror present (“rear-view present”) and four videos without a rear-view mirror (“rear-view absent”) in a random order. Presentation was counterbalanced with respect to road type. For example, if Rural #1 was viewed “rear present” the rear-view footage, the other version of that road type (Rural #2) was presented “rear absent.” This was to eliminate any influence of the presence of the rear-view footage potentially affecting performance differentially across road types. We expected that the combination of both fields of view (“rear-view present” condition) would enhance SA by giving a more immersive experience of the remote scene.

### Participants

Ten participants were recruited randomly online using Prolific (an online participant recruitment tool^4^), meaning the sample was split unevenly by gender, with three quarters (70%) of the participants being female. All participants were United Kingdom residents with English as their first language and possessed a full United Kingdom driving license for a minimum of 3 years. We used bracketed ranges in increments of five to ask participants their age, the number of years they had held a driver’s license and we asked them how frequently they drove (daily, weekly, monthly). We also asked them to record their approximate annual mileage (in 1000 km). Table 1 shows a summary of participant demographic details. Half of the participants drove on a daily basis and only one participant rarely drove. An upper age limit of 75 was set to coincide with United Kingdom driving laws but, during testing, the maximum age bracket selected was 61–65 years old. There were two modal age ranges 31–35 and 41–45. Seven participants drove 5, 000 miles or more in the last year and had 10 years or more driving experience.

**TABLE 1:** Participant demographic lnformation (*n* = 10).

Participant number	Gender	Age	Annual mileage (in 1000 km)	Years held driver’s license	Frequency driving
1	Male	41–45	5–10	21–25	Weekly
2	Female	21–25	<1	<3	Rarely
3	Male	31–35	10–15	11–15	Weekly
4	Female	41–45	1–5	21–25	Daily
5	Female	21–25	5–10	3–5	Weekly
6	Female	61–65	5–10	46–50	Daily
7	Female	26–30	5–10	6–10	Daily
8	Female	31–35	>20	11–15	Daily
9	Female	41–45	<1	21–25	Daily
10	Male	31–35	5–10	11–15	Weekly

### Procedure

Participants were informed that they would see videos of driving scenes and would be asked two questions about the last few seconds of the driving scene. They were instructed to consider themselves the driver and to describe the road that “you” were on, driving maneuvers “you” were carrying out or the behavior of other road users and pedestrians. For the SA Prediction questions, they were advised that they could concentrate on possible future directions “you” may take, the actions of other drivers or road users or the changing physical environment around “you.” We reminded participants to press the “Start recording” button and “Stop recording” button to record their answers after each video. We showed participants an example video (30 s) and played them example spoken answers, recorded by a confederate, in response to the two experimental questions. The example video was from the “rear absent” condition so that they were not primed to the nature of the independent variable before the experimental trials started. Table 2 shows the scripts of the recorded example audio for SA Comprehension and SA Prediction.

**TABLE 2:** Example audio played to participants during the practice trial.

SA Question	Confederate transcript
*Comprehension* ‘What was happening when the video stopped?’	*I’m on a residential road with houses… on both sides and trees…, on both sides… I just went past a big driveway on the left hand side with gates and two …red… cars went past me on the right hand side. I’m just coming up to a signpost …and there is a cyclist on the opposite side of the road coming towards me.*
*Prediction* ‘What will happen next?’	*“The road in front of me was… straight and didn’t have any turns… so I will carry on driving… straight ahead at the same speed… which was 30 mph …and the cyclist will cycle past me on the other side… right hand side of the road.”*

**FIGURE 3:**
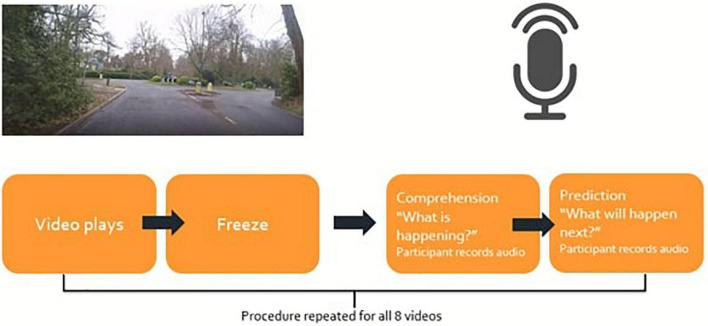
Experimental procedure.

Participants then watched 8 stimulus videos one after another, responding to the two SA questions and the video quality measure after each video (see Figure 3 for a schematic summarizing the procedure). In the debrief, participants were asked “*Do you have any comments about your experience of being a participant in this experiment? For example, was anything unusual or gave you difficultly while you were carrying out the study?”* which was to collect data regarding their viewing experience of the videos.

### Inductive Thematic Analysis of Participant Transcripts

Participants’ verbal responses were recorded as mp3 audio files in Gorilla. The audio files were downloaded into NVivo qualitative data analysis software (QSR International Pty Ltd., Version 11, 2015) and were transcribed by the lead researcher. No participants indicated quality problems that required their results to be excluded.

SA Comprehension and SA Prediction question responses were analyzed separately using the same analysis procedure. All ten participant recordings for each video were analyzed together as one set. An inductive thematic analysis procedure was used to evaluate participants’ naturalistic situation awareness to produce a taxonomy of situation awareness in driving.

Creating classifications to represent driving SA involved extracting information elements from participant transcripts and establishing shared temporal, spatial and semantic concepts between them using inductive thematic analysis. Researchers divided each transcript into singular words and coded each word as an individual item. Similar words or concepts verbalized by participants were grouped together and recorded as sub-themes; for example, the sub-theme “Type of highway” contained the items, “country lane/a country road/a country road/country lane/a rural road/down a country road/road.”

A fundamental objective in coding qualitative responses to stimuli is to make the decisions involved in developing the themes transparent and clearly linked to the aim (Graneheim et al., 2017). The research team, consisting of four people, conducted blind tests to validate the decisions made as to what constituted an item. Each person judged the same five, randomly selected, participant transcripts from different roads and identified the items that were reported in each road transcript. Refinements to the coding scheme were discussed and agreed for each iteration until all researchers were within one item agreement on a final blind test.

#### Reliability Assessment

To judge whether the taxonomy was a robust measure of driving SA that could be used by other researchers, inter rater reliability was assessed. Inter rater reliability establishes the degree to which observers agree on the occurrence of each coded item, in this case, into the separate themes of the taxonomy (Jansen et al., 2003).

To collect a representative sample of the data set, 32 transcripts (16 for each of SA Comprehension and SA Prediction responses to the stimulus questions) across all eight video/road types, signifying 20% of the total dataset, were selected. All road types (8) were represented twice in random order and participant transcripts were randomly drawn with no replacement until all ten participants had been sampled equally, repeating the procedure until 16 transcripts had been selected.

An independent coder was trained in the coding of the taxonomy and their tallied totals for each theme were compared with the lead researcher’s to calculate inter-rater reliability. We adopted a consensus approach to coding, meaning that if raters disagreed they would discuss how to apply the rating scale in that instance, allowing them to come to a decision on how to deal with the conflicting scores (Stemler, 2004).

Cohen’s kappa was calculated for each SA Comprehension theme (6) and SA Prediction theme (4). Cohen’s kappa was appropriate as it controls for chance agreement where raters may be making random guesses if they are not sure which theme an item may fall into. There is general unanimity in the literature that any kappa below 0.60 indicates inadequate agreement (McHugh, 2012). At time 1 analysis, inter-rater reliability between the two raters for each theme was between fair (20–40%) and moderate (40–60%) which represents low agreement and could invalidate the usability of the taxonomy.

On review of the coding tables for each rater, it was found that one tally placed in the “wrong” theme could push two or more themes out of agreement so careful analysis was carried out to discover where the discordance occurred. There were several decisions taken when coding the transcripts as to what constituted an “item”; some verbalizations were ignored, only counted once or moved to a different SA response. The following subsections outline the coding decisions that were made in respect to item definition and inclusion.

#### Treatment of Errors

Two participants (P2 and P6) each had one inaccuracy in their reporting. P2 cited a second roundabout that was not present in the video and P6 claimed that a car had “*flashed lights at me”* when it had not. These errors were not included in the total item list. Similarly, if participants said something that they couldn’t know, as it was not included in the video, we did not include it in the item list, for example,

*“*…*I waited my turn and then proceeded to indicate and go round the vehicle*.” Participant 3

P3 cannot know whether the “driver” indicated or not as this was not shown in the video, so this is not a true SA observation but instead an artifact of schema driven expectations (we “should” indicate when we go round a vehicle). Schemas are discussed in detail in section “Role of Context in Comprehension Illustrated by Use of Schemas.”

#### Coding Repetitions in Participant Transcripts

If participants referred to the same “item” more than once for example a pedestrian but in different contexts, we decided that it should be coded semantically, for example:

*“*… *so I’ve pulled in to let other cars pass me*… *meanwhile the* ***young lady*** *is* ***swinging a bag*** *rather haphazardly* ***has walked past me*** *on the path and walked* ***further up the road*** *and then*… *as I finally manage to drive past the parked cars*… ***she’s there*** *in front of me* ***swinging her bag*.”** Participant 8

The reference to firstly, the *presence* of the pedestrian and their *location* (“walked past me”) is recorded but also their *actions* (“swinging a bag”), *age* (“young”) and *gender* (“lady”) of that pedestrian were each counted as distinct items rather than subsuming these details into a generic “pedestrian.” To gain a full sense of how participants view a driving scene from a remote perspective, every part of their description indicated how they were building up a mental model of the scene and so was included in our analysis. However, repeated mentions of identical information (e.g., the second mention in this example that the person was swinging her bag) were not coded separately.

In the SA Prediction question, some participants suggested more than one possible outcome based on what they had just seen, for example,

“…*there will be either a roundabout or*…*a turn in the road.”* Participant 2.

It was decided to count each of these possible outcomes as valid, because evaluation of the future may produce multiple hypotheses.

#### Irrelevant Commentary in Participant Transcripts

On occasion, participants did not appear to fully put themselves in the ego perspective of the “driver” in the video, commenting that the driving did not reflect what they would have done in a particular situation, for example:

“*I probably waited longer than normal, I would have overtaken and not waited as long normally*” Participant 4

“… *I went from the slow lane to the middle lane*… *for no apparent reason because there is nothing in the slow lane for me to overtake and you should only be in the middle lane when you are overtaking*…*”* Participant 8

As these were not SA observations, these opinions were not coded as items, although the roadway descriptions within them (“slow lane,” “middle lane”) were included in the sub-themes. However, they did illustrate important distinctions between participants and how immersive they found the experiment, which we discuss in further detail in the results section “Understanding of Remote Vehicle State.”

#### Relationships Between SA Comprehension and SA Prediction Transcripts

In some responses, participants did not adhere to the stimulus question and included information that was out of place. Some participants merged SA Comprehension with SA Prediction by referring to future actions (prediction – shown here in bold) in the first question (comprehension – shown here unbolded).

*“I am on a bend turning right and there are cars passing me on the right. To go on the bend, I* ***will be slowing down my speed and being careful to make sure that. be sure because I don’t have much visibility going around the bend.****”* Participant 2

To develop distinct themes in the taxonomy between SA Comprehension and SA Prediction, we removed any references to future actions from the item list for the comprehension category but ensured that they were still included in the SA Prediction category. In the responses to the SA Prediction question, it was agreed that only information in the future tense (as a prediction) would be counted.

#### Item Categorization in SA Prediction Question

It is difficult to make definitive judgments concerning what constitute “reasonable” predictions in driving situations. There are an infinite number of “items” that could be mentioned which make any classification system non-exhaustive. To recognize this, we differentiated between “abstract risks,” which could be any conceivable event and “specific risks” which were named risks that directly related to the events unfolding in the video, as sub-themes within the over-arching theme of “Impending Hazards.”

Also, it is not clear how to account for predictions concerning the absence of items or events, for example:

“*I actually don’t think anything will happen.”* Participant 6

This comment indicates a significant analysis of the absence of risk and would presumably be acted upon accordingly in a real driving situation, however, there is no noun which can be coded as an item. We decided to record this type of response under “Absence of hazards” e.g., no bends ahead/no signs/no pedestrians/no cars present/road clear/nothing will happen.

To re-assess inter-rater reliability of the taxonomy in light of all of these decisions, both the lead researcher and the independent observer were re-trained in the taxonomy. All transcripts were re-coded by both raters, with time 2 analysis exceeding the 80% threshold for almost perfect agreement, producing greater than 85% agreement in all SA Comprehension themes and between 80% and 91% for all SA Prediction themes (McHugh, 2012).

## Results

### Development of a Taxonomy of Situation Awareness in Driving

The sub-themes produced by coding at item level across all road types were pooled to produce a complete list of the items that were mentioned in response to the SA Comprehension and SA Prediction questions. These coded items were grouped into common sub-themes, for example relating to the road/highway or relevant to the driver’s perspective. These sub-themes were collated into over-arching themes which described systematic SA concepts that underpin naturalistic viewing of driving videos. There were 6 over-arching themes in SA Comprehension and 4 over-arching themes in SA Prediction (see Table 3).

**TABLE 3:** Taxonomy of situation awareness in video-relays of driving scenes for SA Comprehension and SA Prediction.

SA Comprehension		

1.1 Road/highway	1.2 Driver’s perspective	1.3 Other drivers/vehicles
***Type of highway*** E.g., country road/lane/main road/city road/dual carriageway/motorway/A road/B road Named road E.g., M3, A30 ***Road description*** E.g., two lanes/single lanes E.g., busy/quiet/long/clear E.g., speed limit ***Type of area*** E.g., village/residential ***Road layout information*** Turn Straight road Junction Bends Uphill/downhill Roundabout (exits 1/2/3) Roadworks/diversions/road blocked Cycle lane Traffic lights Filter lanes Slip road Location of road layout information To the left/right/middle of the road ***Road layout (adjective)*** “sharp” bend, “slight” bend, “very big roundabout” etc., ***Time of day/season*** E.g., dusk/dark winter’s day ***Traffic signs and signals*** On road signs and markings Presence of sign/markings Meaning of sign/markings Location of sign/markings Signpost Presence of sign Meaning of sign Location of sign Light signals Traffic lights (red/amber/green) Level crossings Motorway signals e.g., lane control, fog, information message, camera ***Road “decoration”*** Trees Houses Location of “decoration” Left hand side of the road (driver’s side) Right hand side of the road (offside) Above (hanging)	***Relative position to other cars*** Behind To the right To the left In front/driver following Oncoming traffic on opposite side of the road Passing parked cars Distance e.g., close/far away No cars present No cars behind ***Direction/trajectory of driver*** Going right/left/straight on Position on the road e.g., middle lane, second lane on motorway ***Speed traveling at*** Actual (mph) ***Subjective judgment of speed*** Fast/too fast/slowly ***Driver’s decision/judgment of own action/s*** Temporal judgment (e.g., whether time to pass parked cars) Decision to wait Decision to maneuver out/overtake/nudge’ into oncoming traffic Should slow down ***Rationale of judgment of*** own action/s Significance of obstacles (not enough space for two cars to fit) Why driver is waiting/stationary (waiting for a gap in traffic) ***Driver perception issues*** Lack of visibility Poor light	***Description*** “Vehicle” or type of vehicle (e.g., van/lorry/car etc.,) Many vehicles e.g., “traffic” Make Model Color Registration number Body details (e.g., cages on the side) Identification of company logo (e.g., DPD) ***Direction/trajectory of moving vehicle*** Going right/left/straight on/in left lane ***Speed traveling at*** Actual (mph) Perceived/subjective e.g., “too fast” At the speed limit Queuing traffic/congested Traffic flowing freely ***Purpose of vehicle*** E.g., Delivery/courier, waste/rubbish truck, local authority vehicle, ambulance ***Location of static vehicle/s*** On the verge/On the pavement/Left side/Right side ***State of vehicle*** Parked Abandoned ***Rationale/analysis of other driver action (theory of mind)*** Why driver is slowing down (e.g., driver is looking for an address) Why driver is parked (e.g., driver is going to deliver a package, doing ground works, clearing debris)

**1.4 Dynamic actions of driver and other drivers/vehicles**	**1.5 Pedestrians/other road users**	**1.6 Anticipatory Hazards**

***Maneuver planning/positioning*** Approaching bend Approaching roundabout Approaching traffic lights Waiting Approaching exits Signaling ***Active maneuvers*** Driving straight/Continuing driving Slowing down/speeding up/braking/stopping Going round bend Passing junction/turning Entering/Exiting roundabout (exits 1/2/3) Pulling out/Changing lanes/Pull back into lane Entering roads/motorway Taking exits/coming off Overtaking cars Give way ***Observation*** Checking mirrors Looking for other vehicles Observing other vehicle’s signals (flashing lights, hazards etc.,)	***Presence of pedestrian/other road user*** E.g., “pedestrian,” “cyclist,” “rider,” “dog” ***Location of pedestrian/other road user*** On pavement On side of road ***Location of pedestrian/other road user relative to driver*** On my left On my right Right hand side Left hand side On the other side of the road ***Description of pedestrian/other road user*** Outfit/clothes wearing (e.g., jacket) Color of clothes Holding or carrying specific items (e.g., bag) ***Gender of pedestrian/other road user*** Male/man/boy Female/woman/lady/girl ***Age of pedestrian/other road user*** Young Old ***Action of pedestrian/other road user (what pedestrian is doing)*** E.g., walking. swinging bag ***Purpose of action of pedestrian/other road user (why they are doing it)*** Theory of mind projection E.g., pedestrian stepping into road without looking/driver getting out of car into road without checking for oncoming traffic ***Occupation of pedestrian/other road user*** E.g., local authority worker	***Keeping distance from other vehicles*** E.g., not getting too close, keeping a car length between own car and car in front, being “careful” ***Watching out for other vehicles’ behavior*** E.g., caution before pulling out on a roundabout

**2. SA Prediction**		

**2.1 Changing road environment**	**2.2 Driver future dynamic action/s**	

***Road layout perception*** E.g., bend to the right/road to the left/4 lane motorway ***Environmental markers*** E.g., /railway/hump back bridge/houses/trees ***Traffic signs ahead*** On road e.g., road marking/mph/slow) Sign post e.g., roundabout/motorway/brown sign) Traffic lights (on red/amber/green) ***Changing road details*** E.g., Approaching roundabout/bend approaching/road turning to the right/going uphill/changing traffic lights	***Driver future observation*** Checking mirrors Looking for other vehicles Observing other vehicle’s signals (flashing lights, hazards etc.,) ***Driver future maneuvers*** E.g., Overtake/turn/accelerate/continue to drive/stop/pull out/wait/drive on other side of the road/signal/give way to the right or left/finish maneuvers already started ***Driver future orientation/trajectory*** E.g., Straight on/round the bend/carry on/turn left or right ***Driver future speed*** E.g., mph/faster/slow down/slow down/maintain speed ***Driver position on the road*** E.g., in my lane/on the left hand side of the road/on a bend ***Reaching destination*** E.g., arriving at intended location	
**2. SA Prediction**	

**2.3 Other vehicle/road user predicted dynamic actions**	**2.4 Impending hazards**

***Description of other vehicle/road user*** E.g., one/a number of vehicles/traffic E.g., model/make/color ***Other driver/road user future maneuvers*** E.g., Overtake/turn/accelerate/continue to drive/brake/stop/pull out/wait/drive on other side of the road/signal ***Other driver future orientation/trajectory*** E.g., Straight on/round the bend/carry on/turn left or right ***Other driver future speed*** E.g., mph/faster/slow down/slow down/maintain speed ***Position relative to driver*** E.g., to “my” left/to “my” right/in front/behind/close ***Location of other vehicle/road user*** E.g., on the verge/side of the road/other side of the road ***Purpose of other vehicle/road user*** E.g., industry/occupation	***Driver general caution/awareness*** E.g., looking to see what is coming/being careful/concentrating/wary of distraction/giving wide berth when overtaking ***Specific potential hazards*** E.g., pedestrians/other road users/traffic lights/limited visibility/roundabout Future actions of other road users (e.g., walk in road, cross without looking) ***Location of projected hazard*** E.g., on the right/on the left ***Abstract/hypothetical hazards*** E.g., a crash/animals in the road ***Absence of hazards*** E.g., no bends ahead/no signs/no pedestrians/no cars present/road clear/nothing will happen ***Theory of mind projection to evaluate future risk*** E.g., being in a daydream

The themes, together with the sub-themes within each, were recorded in a taxonomy of situation awareness in driving which encompasses the full range of what participants told us they saw in the videos of the driving scenes. The total proportion of references to each theme in the whole dataset were calculated and can be seen in Table 4, together with the frequency (*n*i) at which each of the sub-themes were referenced within each theme. It is important to note that these frequencies will have been influenced by the specifics of the scenes presented in the videos. Nevertheless, because a wide range of road types and scenarios were represented in the videos, and because many aspects were present in all videos (e.g., the road/highway and the driver’s perspective) we believe that the frequencies of mention for each theme and sub-theme can be informative as long as they are interpreted with caution. The next two sections discuss each of the themes and some examples of sub-themes of the taxonomy. Further examples of each theme with quotes from the transcripts are illustrated in Table 5.

**TABLE 4:** Total proportion of references to each theme and the frequency (%) at which each of the sub-themes were referenced within each theme.

SA Comprehension Macro and micro category list									Total proportion of references

1.1 Road									30%
**Type of highway**	**Road description**	**Type of area**	**Road layout**	**Road layout (adjective)**	**Time of day/season**	**Traffic signs**	**Road “decoration”**		

23.5%	19.6%	5.2%	27.8%	1.7%	0.9%	11.7%	9.6%		

**1.2 Driver’s perspective**									21%

**Relative position to other cars**	**Direction/trajectory of driver**	**Speed traveling at**	**Subjective judgment of speed**	**Driver’s decision/judgment of own action/s**	**Rationale of judgment of own action/s**	**Driver perception issues**			

41.9%	26.3%	5.6%	3.1%	7.5%	12.5%	3.1%			

**1.3 Other drivers/vehicles**									21%

**Description**	**Speed traveling at**	**Direction/trajectory**	**Purpose of vehicle**	**Location of vehicle**	**State of vehicle**	**Rationale/analysis of other driver action (theory of mind)**			

55.2%	4.2%	10.9%	6.1%	12.1%	8.5%	3.0%			

**1.4 Dynamic actions of driver and/or other drivers/vehicles**									23%

**Maneuver planning**		**Active maneuvers**		**Observation**					
**Driver**	**Other driver/vehicle**	**Driver**	**Other driver/vehicle**	**Driver**	**Other driver/vehicle**				

13.7%	0.6%	66.9%	16.0%	1.7%	1.1%				

**1.5 Pedestrians/other road users**									5%

**Presence of pedestrian/other road user**	**Location of pedestrian/other road user**	**Location of pedestrian/other road user relative to driver**	**Description of pedestrian/other road user**	**Gender of pedestrian/other road user**	**Age of pedestrian/other road user**	**Action of pedestrian/other road user (what pedestrian is doing)**	**Purpose of action of pedestrian/other road user (why they are doing it)**	**Occupation of pedestrian/other road user**	

22.9%	5.7%	20.0%	17.1%	11.4%	2.9%	17.1%	2.9%	2.9%	
**1.6 Anticipatory Hazards**							1%

**Keeping distance from other vehicles**	**Watching out for other vehicles’ behavior**						

75.0%	25.0%						

**SA Prediction Macro and micro category list**							**Total proportion of references**

**2.1 Changing road environment**							13%

Road layout perception	Environmental markers	Changing road details	Traffic signs ahead				

40%	9%	36%	15%				

**2.2 Driver future action/s**							43%

Driver future observation	Driver future maneuvers	Driver future orientation/trajectory	Driver future speed	Driver position on the road	Reaching destination		

4%	49%	21%	12%	9%	6%		

**2.3 Other vehicle/road user predicted actions**							25%

Other driver/road user future maneuvers	Other driver/road user future trajectory	Other driver future speed	Position relative to driver	Location of other vehicle/road user	Description of other vehicle/road user	Purpose of other vehicle/road user	

15%	1%	10%	26%	6%	40%	1%	

**2.4 Impending hazards**							19%

Driver general caution/awareness	Specific potential hazards	Location of projected hazard	Abstract/hypothetical hazards	Absence of hazards	Theory of mind projection to evaluate future risk		

23%	41%	4%	15%	15%	2%		

**TABLE 5:** Examples of each theme with illustrative quotes from participant transcripts.

Illustrative quotes derived from the SA Comprehension question categorized into sub-themes of the taxonomy	

1.1 Road/highway	
Traffic signs and signals	“*there was a house on the right-hand side.coming… a sign saying coming… to a left hand bend. Also there was a DPD red van in front of me… past the slow sign on the road, then we came to a built up area with houses so… we slowed down to 30…”* P6 RES#2 WITH
Road “decoration”	*“I was driving along a single lane road, going through countryside…. there were trees on either side of the road and some fences”* P1 A#2 WITHOUT

**1.2 Driver’s perspective**	

Relative position to other cars	*“There were cars to my right but none in the middle lane and there was a lorry ahead in the left lane, where I was but it was quite ahead*.” P2 M#1 WITHOUT
Subjective judgment of speed	*“This was a country road with trees on both sides… I believe we were going a lot faster than 30 mile per hour.”* P6 A#2 WITH
Driver’s decision/judgment of own action	*“So I am sitting there waiting. for the cars to pass on the other side of the road… waiting for a gap… for when I can pull out.” P5 RES#1 WITH*

**1.3. Other drivers/vehicles**	

Location and state of other vehicles	*“and was passing… what looked like an abandoned… van on the other side of the road*.” P8 R#2 WITHOUT
Rational/analysis of other driver action (theory of mind)	*“I think he is trying to find out where to drop his parcel off…”* P5 RES#2 WITHOUT
Purpose of other vehicles	*“some sort of waste truck…”* P9 R#2 WITHOUT

**1.4 Dynamic actions of driver and/or other drivers/vehicles**	

Active maneuvers (driver)	*“I waited my turn and then proceeded to indicate and go round the vehicle.”* P3 RES#1 WITH
Active maneuvers (other driver/vehicle)	*”the black van in front of me had stopped*…*”* P1 A1#WITH

**1.5 Pedestrians/other road users**	

Location of the pedestrian/other road user relative to driver	*“*…*there was a pedestrian walking on the left and there were cars approaching from the right…”* P2 RES#1 WITHOUT
Gender of the pedestrian	*“A lady with a blue shopping bag walked past at the same time.”* P3 RES#1WITH
Action of pedestrian/other road user	*“at one point a local authority worker was in an red*… *orange high vis on the side of the road clearing debris.”* P3 R#1 WITHOUT

**1.6 Anticipatory Hazards**	

Keeping distance from other vehicles	*“Because I’m on a bend and I had slowed down for the vehicle in front…”* P 2 Res#2 WITH

**Illustrative quotes derived from the SA Prediction question categorized into sub-themes of the taxonomy**	

**2.1 Changing road environment**	

Road layout perception	*“To go on the bend I will be slowing down my speed and being careful to make sure that… be sure because I don’t have much visibility going around the bend.” P2 R#1WITHOUT*
Environmental markers	*“*…*the vehicle will slow down to take the hump back bridge over the railway to the right… for… coming in to what I imagine is a small village or town” P3 R#1WITHOUT.*

**2.2 Driver future dynamic action/s**	

Driver future maneuvers	*“I will carry on overtaking the van and then pull back into my lane on the left hand side of the road*…*” P1 RES#2 WITH*
Driver position on the road	*“I imagine that I will continue down the road in the middle lane proceeding past the… vehicles on the left, returning to the outer left hand lane…” P3 M#2 WITHOUT.*

**2.3 Other vehicle/road users predicted dynamic actions**	

Other driver/road user future speed	*“I am coming up behind a van that is going slower than me, so I will have to brake as I come up close behind the van.” P1 A#2 WITHOUT*
Other driver/road user future maneuvers	*“… the Volkswagen golf behind me appeared to be quite close to the vehicle and may attempt to overtake” P3 A#1 WITH*

**2.4 Impending hazards**	

Driver general caution/awareness	*“I am about to drive over a hill… so I predict that I may well encounter… some form of hazard as I… go around the bend.” P7 R#1 WITHOUT*
Absence of hazards	*“I think it will have been just a normal road with traffic on my right hand side… type of a country road… certainly going faster than 30 mile per hour, looking at the video. So, I actually don’t think anything will happen.” P6 R#2 WITHOUT*
Theory of mind projection to evaluate future risk	*”“I’m hoping that she doesn’t step out into the road… so I have to brake suddenly but you never know with people when they are in a bit of a dolly day dream, they might do that…” P8* Res#1 WITHOUT.

*n.b. Notation details as follows: P refers to participant number, each video type is referenced (A#1, A#2, M#1, M#2, RES#1, RES#2, R#1, R#2), WITH/WITHOUT refers to whether the participant viewed the video with rear-view image present, or without rear-view image.*

#### SA Taxonomy Derived From the Comprehension Task

The theme “1.1 *Road/highway*,” was the dominant feature in participants’ descriptions, making up 30% of the total proportion of references in the dataset. Within this theme, the sub-themes “type of highway” (*n*i = 23.5%) and “road layout” (*n*i = 27.8%) contributed the most to participants’ accounts of what was happening in the remote scene. All participants told us about the road that they were currently on, describing the type of road, for example a rural road, but also using adjectives such as “busy” or “fast.” This theme included broad information about traffic signs from either on-road signs or signposts (*n*i = 11.7%). Remote awareness cascaded from detecting the presence of the sign (“*a sign*”), understanding what the sign meant (“*saying coming to a left-hand bend*”) and perceiving the location of the sign relative to the driver (“*past the slow sign on the road*”). This indicates an interplay between the “perception” and “comprehension” SA levels which is discussed in more detail in section “Interplay Between Different Levels of SA.” The nature of the locale in the driving video was also commented upon by all participants in at least one of the videos (e.g., whether it was a village or residential area), with some participants even commenting on trees and road “decoration,” thus building up a vivid narrative of the environment that the car is currently occupying, a crucial requirement for navigation. Although, attention to tangential details such as these might be an example of participant variability in SA which we discuss further in section “Individual Differences in Participant SA,” we suggest that these perceptual encoding details allow participants to construct a narrative of the scene assisting their comprehension.

In the theme “1.2 *Driver’s perspective*,” the driver’s judgment of their own action/s, whether temporal judgments of whether to pass parked cars or wait, was illustrative of the capacity for video relay to transpose space and time allowing the viewer to imagine themselves as the driver in the scene. Although we had not tasked participants to pretend to be an RO, they had been instructed to consider themselves as the “driver” and most consistently referred to “their” relative position to other cars (*n*i = 41.9%) and their current trajectory (*n*i = 26.3%). This suggests that video feed can be a powerful medium when distributing information to ROs. The detail in which participants described “their” perspective suggests that they had a good sense of presence in the scene (although we cannot know how immersive they found watching driving videos) and participants often referenced the speed that they were traveling at (*n*i = 5.6%) on busier road type videos.

The theme “1.3 *Other drivers/vehicles*” demonstrated that being aware of other drivers on the road is an important feature of driving SA as the total proportion of references to what other drivers and vehicles were doing was identical to their judgment of their own action/s in theme “1.2 *Driver’s perspective*” (*n*i = 21% for both). In theme 1.3, participants most commonly mentioned descriptive details (*n*i = 55.2%) of the other driver/vehicle such as make, model and color. We also observed detailed analysis of the presence and location (“*up on the verge*”) but also the state (“*abandoned*”) and purpose (“*some sort of waste truck”/” local authority vehicle”)* of other vehicles. Furthermore, participants attended to the identifying characteristics of other vehicles depicted by logos or branding on the side of the vehicle, for example in the video, Residential #2, the “driver” is following a DPD van which all participants alluded to in their verbal accounts. These data were used to conclude that it was likely that the driver would make frequent stops as they were “*looking for somewhere to park up to deliver a parcel,”* showing that theory of mind analysis (*n*i = 3%) of other road users’ ongoing behavior is a component of building driving SA.

Both the driver and other drivers’ driving operations featured heavily in the qualitative descriptions of what was happening in the driving videos, highlighted in the theme 1.4 *Dynamic actions of driver and/or other drivers/vehicles.*” This theme was easily separated into stages, where “drivers” were planning maneuvers (*n*i = 13.7%), carrying them out (*n*i = 66.9%) or making observational checks (*n*i = 1.7%). Participants’ nuanced awareness of the dynamic driving process was evidently a key feature of their attentional focus, even from a remote viewing position and, in less detail, they also commented on the dynamic actions of other drivers.

We also found that consistent features of information such as presence, location and characteristics were reported in the theme “1.5 *Pedestrians/other road users*,” in a similar style to how participants told us about both road signs and other vehicles in other themes. Participants noted the *presence* of a pedestrian (*n*i = 22.9%) if there was one in the video, as well as reporting *where* they were within the remote scene relative to the driver themselves (*n*i = 20%). Participants frequently provided a physical description (*n*i = 17.1%) and what activity they were engaged in (*n*i = 17.1%) and the gender of the pedestrian (*n*i = 11.4%).

Hazard perception has been identified in SAGAT measures as belonging to Projection thus restricted to responses concerning the prediction of future events, yet we found evidence that the theme “Anticipatory hazards” firmly belonged in the SA comprehension taxonomy section. Recent analysis of freeze and probe methods such as SAGAT has suggested that the debate surrounding SA is mainly concerned with how to measure SA effectively, instead it should be focused on whether the levels can truly be considered separate (de Winter et al., 2019). Although this theme represented only 1% of the total dataset, it was evident that participants had not rigidly stuck to the “comprehension/prediction” distinction including hazards such as keeping distances from other vehicles (*n*i = 75%) in their responses to the comprehension question, suggesting that conceptualizing driving SA into three separate levels as suggested by Endsley may be an oversimplification. We discuss this further in section “Interplay Between Different Levels of SA.”

#### SA Taxonomy Derived From the SA Prediction Task

Some participants were not confident at making predictions or found the question facile. Other participants took a far more calculated approach to assessing the situation that had occurred at the end of the video and made predictions, which logically continue from the last action/s or were derived from micro cues, such as where someone’s head is turned (not just whether they are signaling in that direction). There were strikingly different strategies to predicting – carefully judging from current information how the scene can play out in the future or being prepared for any eventuality. It is impossible to say which is the better approach, as being too sure of what will unfold in the next few seconds may make you “blind” to an immediate hazard that presents itself without warning. Participant transcripts revealed that predictions are constructed from expectancies and experience, other drivers’ actions, knowledge of the rules of the road and the likely behavior of other drivers.

Furthermore, in the theme “*2.1 Changing road environment*” all participants demonstrated an evaluation of whether the road would stay the same or change in the future (*n*i = 36%) – this is clearly an important consideration that also shapes the assessment of risk and future maneuvers. For example, if you are going to go around a bend you will expect to slow down and be conscious of possible hidden obstructions. This type of driving is sometimes known as defensive driving, which draws on anticipatory responses to current driving states rather than relying on the situational model (Walker et al., 2009).

The theme, “*2.2 Driver future dynamic action/s*,” dominated the total proportion of references in the data set (43%). Finishing maneuvers that had been started when the video ended, such as pulling back into the lane on the driver’s side of the road were regularly cited (*n*i = 49%). In some of the road types such as the motorway videos, participants also told us about future positioning on the road, for example which lane they would be occupying. We also saw temporal references whereby participants were analyzing the spatial distances that they were physically able to traverse based on their remote estimation of speed. For example, Participant 4 decided that there was enough time left on the “green” light to get through. If they were remotely controlling the vehicle, they may have put their foot on the accelerator to ensure this happened, but they may have been mistaken. This would be an important consideration for a RO. If their temporal judgment were negatively affected by the remote location, this could have dangerous consequences.

In the theme, “*2.3 Other vehicle/road user predicted dynamic actions*,” participants framed their future actions in the context of other road users, revealing an awareness that their future maneuvers did not happen in isolation. Description of the other road user (*n*i = 40%) and their relative position to the driver (*n*i = 26%) were the main subjects of this theme showing perceptual and spatial awareness of the total road environment was being drawn to make predictions about what would happen next.

Participants mentioned specific potential hazards (*n*i = 41%) far more frequently in the SA Prediction question than in the SA Comprehension question which was recorded in the theme, “*2.4 Impending Hazards*” although this theme contributed only 19% to the total dataset. An ambiguous sense of “being careful” was a regular consideration shown by all participants in response to the SA Prediction question. Interestingly, the *absence* of stimuli was also noticed and commented on (*n*i = 15%) as often as hypothetical hazards (*n*i = 15%), for example if there is no sign warning for a bend, we can assume that it is a straight road ahead thereby eliminating consideration of the potential hazard of a sharp bend.

### Qualitative Findings Relating to SA in Remote Driving Contexts

In addition to developing the taxonomy, we were also able to interrogate participant transcripts to identify patterns that were apparent in the construction of remote SA of driving videos. The following section focuses on the overarching patterns that surfaced during the analysis of participant transcripts which illustrate the complexity of building SA in remote scenes.

#### SA of Participants in Relation to Absence or Presence of Rear-View Information

One aspect of our investigation was to compare SA of participants in the presence and absence of rear-view information. Some participants appeared to incorporate the rear-view information into their SA, either through indirect references to perceptual information “behind” them or to predict what the car following them may do next. However, no clear effects of this manipulation were evident in the transcripts and qualitative comments in the Debrief showed disagreement about whether a rear-view mirror was useful or was ignored completely (shown in Table 6).

**TABLE 6:** Attitudes to the presence or absence of the rear-view mirror in the videos as indicated in the debrief.

Participant comments in the Debrief concerning the rear-view mirror	

Participant 6	*“I liked the rear-view camera as people should be using it more. I found that I still used it and also can keep eyes on the road for more fullness.”*
Participant 1	*“When the rear-view image appeared the first time it confused me as I didn’t know what it was so I ignored it. When it appeared the second time I paid more attention and realized what it was and I definitely gave a more detailed description and remembered more.”*

Participant 9	*“I don’t think I looked at the rear-view camera as I wanted to concentrate on the road ahead. There was only one point that I recall looking at the rear-view camera and that was when “I” was stopped behind a parked car to see how many vehicles were stopped behind me (and drivers potentially getting grumpy if I took too long to move).”*

#### Individual Differences in Participant SA

There were noticeable differences between participants in what they reported from the driving videos even though they all saw the same scenes. Some participants were descriptive and some extremely succinct, for example, Participant 7 reported 90% less than Participant 5. To illustrate this point, in the video Residential #1, some participants merely described the presence and location of the pedestrian, whereas others gave additional details about her gender *(“lady*”), age (“*young*”) and what she is carrying (“*shopping bag*”). This could be evidence of individual speaking style, whereby some people use enhanced linguistic codes to provide “*verbal elaboration of meaning”* so some of the differences may relate more to speaking style than actual underlying awareness (Bernstein, 1964, p. 630). Other discrepancies in the verbal reports relate to differences in underlying awareness and subjective judgments as what is important in the scene. For example, a salient feature of video R#2 is an abandoned refuse truck parked on the verge by the side of the road (as the driver may emerge later down the road which could present a hazard) but a minority of participants neglected to mention its presence at all.

There were also noticeable differences between what participants observed in the driving videos. Some participants focused almost exclusively on road features such as road layouts and trees, whereas others reported speed limits very consistently. These differences between participants’ attention and reporting styles appeared to remain fairly consistent within participants – someone who observes types, makes and colors of vehicles in one road makes similar observations on all the videos shown, whereas someone else never alludes to these details at all, but instead shows consistent attention to the location or purpose of other vehicles. For example, only three participants referenced details of “road decoration,” a sub-theme in “1.1 *Road/highway*,” but they did so consistently in every video which contained houses or trees. This demonstrates potential participant variability in building SA in remote contexts.

#### Understanding of Remote Vehicle State

Participants made frequent subjective estimations to the speed that “they” were traveling at, which tells us even through second-hand, indirect cues (the monitor view) they had a sense of traveling at speed.

*“This was a country road with trees on both sides. I believe we were going a lot faster than 30 mile per hour.” P6 A#2 WITH*

However, in some cases (including this example) their beliefs were incorrect, as the videos were filmed at the exact speed limits (or less dependent on traffic conditions) of each road.

Awareness of the spatial limitations of the car was also evident. For example, in video Residential #2, the “driver” is waiting behind a queue of parked cars until there is a gap in traffic, as the road is not wide enough for two cars to pass, and all participants demonstrated understanding of this restriction.

*“I have to come to a stop because there are cars parked on my side of the road. and that means that there is only one side of traffic that can get along the road. So I am sitting there waiting. for the cars to pass on the other side of the road. waiting for a gap. for when I can pull out.” P5 RES#1 WITH*

Although higher levels of precision would be required in order for an RO to maneuver the vehicle safely in contexts such as these, the general principles of driving and knowledge of road size may be employed to make practical SA decisions in remote contexts relating to three-dimensional navigation.

#### Theory of Mind Analysis in SA Comprehension and SA Prediction

Being aware of other drivers on the road is an important feature of SA and safe driving. Participants allocated the same proportion of the time to reporting what other drivers and vehicles were doing as they did to describing their own maneuvers. Being able to predict other people’s behavior by explaining their actions as a product of their independent mental state is known as having a theory of mind (Gallagher and Frith, 2003). Participants demonstrated theory of mind (TOM) in both SA Comprehension (1.3 Other drivers/vehicles: Rationale/analysis of other driver action’) and SA Prediction (2.4 Impending hazards: Theory of mind projection to evaluate future risk) when trying to analyze the reasons for other drivers their behavior (see Table 5 for quotes in each of these themes).

Although the frequency of these references was low (only 3% in 1.3 Other driver’s/vehicles category and 2% in 2.4 Impending hazards) this sub-theme is highly relevant to interpreting how RO SA is constructed. An important theoretical consideration in thematic analysis is that crucial themes may have few occurrences, yet contribute toward a greater understanding of the behavior or phenomenon (Braun and Clarke, 2013). Although rare, participants were seeking a rationale/analysis of other drivers’ actions, such as why the driver is slowing down (e.g., looking for an address) or parking (e.g., driver is going to deliver a package). TOM analyses were also important in relation to pedestrians, for example when considering whether they were likely to step into road without looking. The importance of interpreting the intentions of other road users for achieving effective SA cannot be underestimated in remote contexts.

#### Role of Context in Comprehension Illustrated by Use of Schemas

Endsley (2000) included schema as an integral part of the mental model triggered to develop comprehension and prediction of the scene even though misapplying information or filling in missing information can lead to mistakes or prediction errors. For example, in the video A #2, one participant commented in the SA Prediction transcript that, because she saw an ambulance earlier in the video, she was expecting to see an accident on the road ahead which did not materialize.

Participants in our study also made use of context to expand their comprehension of what was happening in the driving scene drawing on generalized “schemas” of driving to describe actions that they could not have “known” they were doing from the video, such as checking mirrors and signaling:

*”*… *I was just stuck there for a long period of time waiting to indicate to go round some vehicles parked on the left hand lane. A lady with a blue shopping bag walked past at the same time. I waited my turn and then proceeded to indicate and go round the vehicle.” P3 RES#1 WITH*

Deriving context may enhance comprehension when viewing videos of driving scenes as it can be used to make predictions about the likely actions of other vehicles or road users. Although the type of area in 1.1 Road/highway was only mentioned 5.2% of the time in the total dataset, for the video Residential #1 it was described by all participants. This may be because this detail provides information about the likelihood of pedestrians being present, that traffic may be more congested, even that schools may be in the locale. These same mental models would be unlikely to be produced in rural areas so, comparatively, this experiential data may feed into the SA process in some contexts more than others.

#### Parallel Processing in Building SA of Video Driving Scenes

Our analysis uncovered evidence that a complex range of feed-forward and feedback processing is engaged to acquire SA of video driving scenes. We can see this process in the following excerpt,

*“I’m hoping that she doesn’t step out into the road. so I have to brake suddenly but you never know with people when they are in a bit of a dolly day dream, they might do that. so I will continue along the road not only looking at cars parked in front of me and cars coming the other way so there is only room for one car to pass the parked car. but also keeping an eye on this young lady until I have passed her.”* Participant 8

Awareness of the pedestrian (SA Perception) is used to predict expected outcomes (“*step out into the road”*) (SA Prediction), incorporating theory of mind (“*when they are in a bit of a dolly daydream*”) (SA Comprehension) and drawing on gender stereotyping and other schema-based reasoning. At the same time, the participant is constructing a narrative of a likely future (“*keeping an eye on this young lady*”) which also draws on further perceptual processing (age of the pedestrian “*young*”) which again may be used to estimate the likelihood of the event occurring (SA Comprehension/SA Prediction). This implies that SA in driving involves parallel processing, whereby SA Comprehension and SA Prediction can be developed simultaneously, rather than the serial progression through the levels that is suggested by previous models.

#### Interplay Between Different Levels of SA

SA has, thus far in Psychology, been regarded as the *total amount* of information in our possession about our environment, but how these information points relate to each other is just as important, as an interaction between the different levels of SA occurs to develop the picture of the scene (Walker et al., 2009). Endsley (2017b) maintains that the levels of SA are ascending, for example, perception feeds in to comprehension, however, we found evidence that a “higher” level can feed downward to a “lower” level. SA Comprehension or SA Prediction may influence perception; “drivers” may perceive something because of a contextual detail that they had comprehended earlier. For example, participants may be making sense of the highway layout,

”*A single carriage road. speed limit. I think was 50 miles per hour. so although there were single lanes. there were quite a lot of. filter lanes for turning to the right.”*

But then recognize the filter lanes as they pass them, demonstrating SA perception being fed by comprehension,

*” so I had just passed two of those filter lanes to turn to the right.”* Participant 9

Here perception is seamlessly integrated into comprehension in both a feed-forward and feedback association. This exemplifies the clear interplay between perception and comprehension SA in driving.

In driving, estimating and analyzing hazards is important, particularly in relation to identifying driver adjustments that are necessary to prevent potential hazards from developing into actual hazards (for example leaving adequate stopping distances or slowing down and looking for oncoming traffic). Hazard perception has been identified in SAGAT measures as belonging to Level 3 Projection (see also Horswill and McKenna, 2004) and therefore restricted to responses concerning the prediction of future events. Our research instead suggests that elements of prediction are embedded in building comprehension of a remote scene. This can be seen in the example below,

*“I am on a bend turning right and there are cars passing me on the right. To go on the bend, I will be slowing down my speed and being careful to make sure that. be sure because I don’t have much visibility going around the bend.”* P 2 (“Rural #1”).

Although there may have been a perceptual trigger in the immediate environment to prompt an expectation that something may develop in the future (such as a road bend), the projected hazard (lack of visibility) is not yet immediately located in the scene, which challenges the temporal nature of Endsley’s model, which states that SA is

*“the perception of the elements of the environment within a volume of space and time, the comprehension of their meaning, and the projection of their status in the near future”* (Endsley, 1988, p. 792).

Instead, an emerging scene in the “now” ties to “future” predictions through a feedback and feed forward process. In summary, separating driving SA into entirely distinct levels may oversimplify the nuanced understanding of the remote scene required to build up, maintain and update SA when drawing information from remote contexts.

## Discussion

### Comparison With Previous Research

The current study took a novel approach to measuring SA in remote driving, employing a qualitative verbal elicitation task to uncover, in its most basic form, what people “see” in a remote scene when they are not constrained by rigid questioning. Participants provided an abundance of information and qualitative detail enabling the construction of a taxonomy encompassing the types of information that are typically derived from a remote scene.

Previous models of SA have suggested that experts may have better ability than novices in building effective SA (for example hazard perception has been shown to be faster for experienced drivers compared to novice drivers (Huestegge et al., 2010; Gugliotta et al., 2017)), but few have investigated the variability between individuals in *how* they derive situation awareness in a scene, what information they sample and how much importance they give to different pieces of information in the environment. In this study, we observed differences between participants whereby, for example, some focused almost exclusively on road features (e.g., road layout, trees) whereas others reported speed limits very consistently. These patterns of attention and reporting style appeared to be consistent *within* participants – someone who observed types, makes and colors of vehicles in one video tended to make similar observations on all the videos shown, whereas someone else may never allude to these details at all, but instead direct attention to the location or purpose of other vehicles for each video.

No clear effects of the manipulation between presence and absence of rear-view information were manifest in the transcripts. Studies which have used eye tracking to measure the attention participants have given to the road ahead and the mirrors whilst viewing driving videos report a decrease in glance frequency to the rear-view mirror over time (Unema et al., 2005; Over et al., 2007; Lu et al., 2017). It is possible that participants had been accessing the rear-view information during the video but had not used the rear-view mirror recently. If they did not volunteer any information relating to that view in response to either SA Comprehension or SA Prediction questions, even though they had looked at it during the video, the qualitative measures used in this study would be unable to uncover this behavior. We may have found that participants struggled more in the absence of the rear-view mirror if they had been required to do anything active in the scene rather than passively describing it.

We note that, in addition to the provision of a rear-view feed, designers of interfaces for ROs of AVs are likely to end up providing a sophisticated data overlay including multiple sources of information about the car and the road environment to assist in the creation and maintenance of SA. Given that this is such a new area of research, we focused here on the role of video relays in developing awareness of a remote scene. Current research in our laboratory is investigating the role of both rear view and auditory feeds in supporting the development of remote SA (see Mutzenich et al., 2021, in preparation) and future work to investigate the impacts of additional information overlays will also be essential.

Endsley’s original approach implied a clear separation between the three SA levels and has been criticized by many researchers for this distinction (see Sorensen et al., 2011; Salmon et al., 2012). More recently, Endsley has dismissed this characterization as a “fallacy” and acknowledges a high level of integration between the different levels, arguing that the three levels are “*ascending*”; you can go back and search for perceptual data to back up comprehension and projection (Endsley, 2015, p. 8). However, the quantitative nature of SAGAT queries forces a starker distinction between the levels in order to enable researchers to score them separately, implying that perception can be assessed separately from comprehension and so on.

The qualitative methodology used in this study allowed a freer and less constrained investigation than is possible using the quantitative SAGAT-based metrics. For example, although the transcripts were analyzed separately for SA Comprehension and SA Prediction questions, participants typically appeared to blend this information organically; for example, awareness of “hazards” was apparent in both questions, suggestive of the fact that that building up SA in driving contexts is a flexible and fluctuating process of combining comprehension and prediction globally rather than serially, as has sometimes been implied by previous SAGAT methodologies (Jones and Endsley, 1996; Endsley, 2000, 2017b). In addition, the individual variability in reporting detail of the scene is likely to be missed by SAGAT paradigms. To our knowledge, no other research to date has directly measured how SA is freely constructed in driving contexts.

Additionally, a novel and positive aspect of the naturalistic qualitative analysis used was the ability to extract unexpected information about what participants see (or don’t see) in remote driving scenes, which is not possible using approaches in which participants are constrained by questions. The sub-theme *“Absence of hazards”* in the SA Prediction theme “2.4 *Impending Hazards*” illustrates that anyone reporting “no risk” would receive a score of zero on a SAGAT test because no specific items or events are mentioned, yet this is exactly the type of careful data gathering from the remote scene that would be desirable in an RO and would constitute accurate prediction. Similarly, the sub-theme, *“Subjective judgment of speed* e.g., fast/too fast/slowly” in the Comprehension theme “1.2 *Driver’s perspective*” would also be missed by SAGAT as although queries may ask participants to report actual speed (mph) there is no insight into whether the participant has judged that speed to be appropriate. The taxonomy illustrates myriad observations, calculations and adjustments required in driving scenarios which have gone unnoticed in other research paradigms. This type of thorough understanding of what SA comprises in remote contexts is essential before we can start to make judgments of what “good” SA may look like and assess whether quantitative metrics can accurately judge whether or not someone has good SA.

### Limitations of Research

There may have been issues related to the sample of participants used in our study. We did not issue a test on Highway Code to participants prior to the study, so there may have been a variable base level of knowledge between them. However, there were no clear individual or subgroup differences apparent which may have influenced what people spoke about, thus influencing the data that fed into the taxonomy and there was no evidence to indicate that their performance would not be mirrored in another group. In addition, although the sample size of participants (*n* = 10) was small, the total transcripts in response to the video prompt questions amounted to over 8,000 words which presented a rich and diverse dataset.

Another potential limitation concerns the possibility that the use of an example video depicting the “rear absent” condition at the start of the study may have discouraged participants from providing rear view information in response to future videos where this information was present. This seems unlikely because all participants mentioned information at some point that was present in the rear-view feed.

A further consideration is that in this task participants were not actively driving and so were not subject to attention being diverted by other driving tasks such as changing gears, depressing pedals and other peripheral distractions (e.g., changing music). Viewing remote video driving scenes may add a sense of speed or at the very least, distort the viewers’ sense of motion. Without force feedback pushing you back into the seat or information from the tire friction on the road, it is difficult to accurately judge how fast you are driving and to remain engaged in the driving task (Tang et al., 2013). Although the results cannot represent authentic driving experiences, ROs are unlikely to be carrying out these tasks either, so from that perspective this study may not differ greatly from the task of an RO. Mackenzie and Harris (2015) showed that participants were slower to detect hazards when driving themselves as opposed to passively viewing a video, so RO SA may be augmented by the detached nature of their location and the singular nature of their task – to work out from the driver’s perspective what the problem is, via second hand information from the scene.

Some themes in the taxonomy were less prevalent than others, but this may have reflected the specifics of the videos used, rather than the overall nature of the participants’ SA itself. For example, theme “1.5 *Pedestrians/other road users*” represented only 5% of the total data set but this could be because very few of the videos contained images of pedestrians and other road users, as the videos were predominantly filmed on busy highways and motorways, yet all participants mentioned pedestrians when they were present in the video. In addition, the taxonomy did not include any sub-themes relating to roadworks which would be highly relevant to remote operators (as outlined in the introduction to this paper, common challenges to AVs are deviations from their path required by construction works which may be signaled by workers using hand gestures). The naturalistic element of the stimuli used in this study meant that no roadworks were encountered while the videos were being filmed. Future stimuli should incorporate a wider range of driving scenarios, such as busy urban streets, to enable the further generalization of the themes identified. There is also scope for future research to test some of the smaller sub-themes to see if they are replicated by future observers.

For example, the sub-theme *“Theory of mind”* in both SA Comprehension and SA Prediction taxonomies did not feature in many participants’ responses yet presented an important finding in relation to how SA is constructed in a “sense making” context. In thematic analysis methodology, researchers must use their judgment as to how many occurrences of an item are necessary for it to be important (Braun and Clarke, 2013). Indeed, we propose that TOM is a critical element involved in processing and comprehension of a driving scene. Spiers and Maguire (2007, p. 1675) also found examples of TOM considerations (which they termed “spontaneous mentalizing,” referring to participants thinking about the thoughts of others) in their study of London taxi drivers engaging in a verbal description protocol whilst watching/playing a realistic first-person driving game in an MRI scanner. SA Comprehension may require frequent transitions from the driver’s perspective to imagining another driver’s intentions, then acting on that information. Thus, it will also be necessary for ROs to practice TOM if required to engage dynamically with the remote driving task. Future stimuli should include more direct interactions with other road users and other vehicles to ascertain whether this sub-theme would have more prevalence in these driving situations and give more support to its inclusion in the taxonomy.

As the study was hosted online, it was necessary to present the videos in a low resolution (811 × 456) to enable them to be cached and streamed using the hosting site Gorilla.sc. Although ROs are likely to receive higher resolution video and on a larger format than desktops or laptops there was no evidence from participants’ performance feedback in the debrief to suggest that the quality of the display was insufficient for them to extract the necessary information. However, it would be beneficial in future studies to set a standard screen size to give a broader and more HD viewing experience which may make it more immersive or make use of VR to present the visual information as if the driver were *in situ*.

The use of inductive thematic analysis methodology involved an element of subjectivity when classifying and grouping themes. Future research may wish to extend this work with potentially more objective analyses. For example, it has been suggested that network analysis can be used to model SA, by identifying first the information underpinning SA such as “noun-like information elements” and then establishing relationships between different pieces of information (Walker et al., 2009, p. 680; Salmon et al., 2013).

### Conclusion and Recommendations for Future Work

There are several projects currently underway in the United Kingdom designed to understand a range of aspects of remote operation, including human factors considerations, with the aim of enabling remote operation to become a feasible transport opportunity. This research contributes toward the knowledge that will enable the acceptance of autonomous technology in the future.

More specifically, adopting a more nuanced approach to considerations of situation awareness could improve the design of remote operation support systems. Our research suggests that designers of interfaces for ROs should take careful consideration of the scope and range of the information derived from the remote driving videos and also the variability between participants in how they construct situation awareness of remote scenes. We recommend engaging in iterative research processes before and after implementing new graphical user interfaces to ensure that ROs are given information that has been empirically proven to be useful in their SA development. This knowledge may also contribute to the further development of industry standard taxonomies for remote operation so that regulatory frameworks can be established with regards to the training and technology necessary to carry out the role.

Another interesting use of the taxonomy would be to determine which sub-themes are indispensable in remote SA and which are “nice to have,” from the perspective of the actions of remote operators. For example, in the theme “1.3 Other vehicles/road users,” having awareness of the make, model, color etc., of vehicles on the road around you may not be as crucial as understanding the location or trajectory of the vehicles around you on the road. Yet, if there was a crash incident, details such as these would be important for the identification and reporting of other vehicles at the scene. Determining which are the minimum requirements for remote operator SA will be important for selection and training of ROs in the future.

The evidence in this study provides a rich catalog of verbal data that exemplifies the interactions between different SA levels that operate when participants process information from a remote naturalistic driving scene. Our open-source videos of remote driving situations can play a role in developing further understanding of the unique SA requirements for ROs, supporting the construction of new SA questions based on the information that participants in this study have been shown to extract from the videos. The proposed taxonomy can also be used in future empirical work to design queries that can effectively measure RO SA. This work is currently underway in our lab.

## Data Availability Statement

The original contributions presented in the study are included in the article/supplementary material, further inquiries can be directed to the corresponding author/s.

## Ethics Statement

The studies involving human participants were reviewed and approved by Royal Holloway, University of London Psychology. The patients/participants provided their written informed consent to participate in this study.

## Author Contributions

CM wrote the manuscript. PD, SD, and SH reviewed several drafts and contributed to revisions of the manuscript. All authors approved the final manuscript.

## Conflict of Interest

The authors declare that the research was conducted in the absence of any commercial or financial relationships that could be construed as a potential conflict of interest.

## Publisher’s Note

All claims expressed in this article are solely those of the authors and do not necessarily represent those of their affiliated organizations, or those of the publisher, the editors and the reviewers. Any product that may be evaluated in this article, or claim that may be made by its manufacturer, is not guaranteed or endorsed by the publisher.

## Footnotes

1
https://github.com/clareyclare/clareyclare/blob/main/video-relays%20of%20driving%20scenes

2 www.techsmith.com, Version 2018. 0. 6.

3
www.gorilla.sc

4
www.prolific.co
